# Screening of the Leaf Extracts of Culinary Herbs (Apium graveolens, Petroselinum crispum, Cichorium endivia, and Anethum graveolens) for Their Antibacterial Activity Against Escherichia coli

**DOI:** 10.7759/cureus.54242

**Published:** 2024-02-15

**Authors:** Prabhat LNU, Jagriti LNU, Sushil Kumar, Akash Bansal

**Affiliations:** 1 Biochemistry, All India Institute of Medical Sciences, Gorakhpur, Gorakhpur, IND; 2 Biochemistry, All India Institute of Medical Sciences, Patna, Patna, IND

**Keywords:** drug resistance, in vitro phytochemicals, e. coli, escherichia coli, antimicrobial herbs, antibiotics therapy

## Abstract

Introduction: In the current era, infectious diseases pose a significant global challenge, primarily attributed to the widespread and prolonged use of antibiotics, which develop antimicrobial resistance. A significant proportion of pharmaceutical agents utilized globally can be traced back to plant origins, constituting approximately 25%. Medicinal applications harness a wide spectrum of plant-derived components, including flowers, leaves, stems, fruits, roots, waxes, oils, bioactive compounds, phytochemicals, and various other constituents.

Materials and methods: Our experiment evaluated the antibacterial activity of four different culinary plant leaf extracts. These extracts were prepared using four different solvents and were investigated against the gram-negative bacteria *Escherichia coli DH5α* using agar well diffusion and agar disc diffusion methods by measuring the zone of inhibition.

Results: The aqueous extract of all leaves did not show any antibacterial activity, likely due to poor diffusion due to the formation of a precipitate. Conversely, *Cichorium endivia* has shown the highest antibacterial activity in isopropanol as compared to other herbs. Among the herbs examined, organic extracts from endives and soybeans have demonstrated notably strong antibacterial activity compared to the other herbs.

Conclusion: Conducting a systematic screening of leaf extracts from various culinary herbs to assess their antibacterial effectiveness against *E. coli* has produced encouraging and noteworthy results. In the investigation of various herbs, organic extracts derived from endives and soybeans have exhibited particularly robust antibacterial efficacy when compared to other herbal extracts.

## Introduction

Antibacterial leaf extracts have garnered considerable interest for their potential applications in practical settings. These extracts, derived from plant leaves, contain bioactive compounds with antibacterial properties, making them promising candidates for various uses. Antibacterial leaf extracts can be incorporated into traditional or alternative medicine practices for treating bacterial infections.

The major problem with synthetic antibiotic is that it incurs antimicrobial resistance. The presence of this resistance makes antibiotics and other antimicrobial therapies ineffective, creating difficulties in treating infections and increasing the chances of disease spread, serious sickness, disability, and death [1]. This highlights the crucial significance of thoroughly tackling and comprehending the consequences of antimicrobial resistance with its influence on death rates and healthcare results [2].

The Rig Vedas, and ancient texts, report the use of natural products to treat several diseases of humans as well as animals, a practice now recognized as Ayurvedic medicine. Twenty-five percent of all drugs used worldwide have their origins in plant sources [3]. Various parts of plants and their products such as flowers, leaves, stems, fruits, roots, waxes, oils, bioactive components, phytochemicals, and many more are used for medicinal purposes [4].

To address these challenges, a naturally occurring compound from medicinal herbs with effective antimicrobial properties without any negative effect on health is very much required. About 120 plant-based drugs are made from just 95 plant species only [5]. The phytochemical compounds present in the leaves of these herbs are likely to inhibit the growth of bacteria by targeting the biosynthesis of bacterial proteins, destruction of cell membranes, cell wall synthesis, and inhibiting the replication machinery and metabolic pathways [6].

Culinary herbs, namely, *Apium graveolens*, *Petroselinum crispum*, *Cichorium endivia*, and *Anethum graveolens,* were used in this study.

Apium graveolens

It is commonly known as celery and belongs to the family of Apiaceae. This is a biennial herbaceous plant native to Mediterranean regions like Asia, Africa, and Europe. In India, it is found in the Northwestern Himalayas, Punjab, Haryana, and Western Uttar Pradesh. Due to the presence of essential oils (d-selenene, sedlanolide, and sedanoic acid anhydride), it provides good culinary taste and flavor to food. In the Ayurvedic system of medicine, it is used to cure colds, flu, fluid retention, indigestion, and arthritis. It has antioxidant, anti-inflammatory, antispasmodic, antibacterial, anti-fungal, anti-cancer, and sedative properties [7]. Also, it is used for stomach pain and jaundice and helps to increase the production of breast milk. It is useful to treat diabetes and exhibit antibacterial activity against *Escherichia coli*. It also has anti-cancer components like polyacetylens and phthalide which help to detoxify carcinogens [8].

Petroselinum crispum

It is commonly known as parsley and belongs to the family of Apiaceae. This is also a biennial herbaceous plant, native to central and eastern Mediterranean regions [9]. In India, it is found in Jammu and Kashmir, Punjab, Uttarakhand, Uttar Pradesh, Maharashtra, and Karnataka [10]. It is used as an additive and flavoring agent in food products and perfumes. It cures gastrointestinal disorders, kidney stones, hypertension, and hepatic disorders, reduces oxidative stress, and treats skin diseases. It has antioxidant, antibacterial, anti-fungal, immunosuppressant, anti-ulcer, and estrogenic properties. Due to the presence of essential oils myristicin (phenylpropene) and apiol, it shows antioxidant properties and helps to increase estrogen production. It also has carotenoid substances such as neoxanthin, β-carotene, lutein, and violaxanthin [11]. It exhibits antioxidant activity through increasing lipid peroxidation and anti-platelet activity due to the presence of aglycone flavonoids. It also has analgesic effects and protects against hepatotoxicity, nephrotoxicity, and proliferative activity in estrogen-sensitive breast cancer cell lines [12].

Cichorium endivia

It is commonly known as endive and belongs to the family of Asteraceae. This is a perennial herbaceous plant, native to Mediterranean regions of Europe, Western Asia, and North America [13]. Due to the presence of the high content of healthy components, it can help to cure diabetes, jaundice, malaria, gallstones, hemorrhoids, and anemia. It exhibits prebiotic action due to the presence of fructans and anti-cancer properties by inhibiting tumor cell growth and retard aging. It has a low glycemic index, which is recommended for diabetic patients [14]. It protects against sunburn and UV-induced pyrimidine dimer formation and IL-6 expression to prevent erythema [13].

Anethum graveolens

It is commonly known as dill (soya) and belongs to the family of Apiaceae. This is an annual herbaceous plant, native to Mediterranean regions, Southern Russia, Central Asia, and Southeast Europe. It is used to treat colic pain in babies, increase appetite, and cure urinary problems, piles, stomachache, indigestion, and flatulence [15]. It is used for flavor and as a preservative in food products. It shows antimicrobial, anti-inflammatory, analgesic, and gastric mucosal protective effects and increases progesterone. It also exhibits antibacterial activity and decreases inflammation and pain [16].

In this study, we have done an experimental analysis of the antibacterial properties of aqueous, ethanol, methanol, and isopropanol extract of *Apium graveolens, Petroselinum crispum, Cichorium endivia, *and *Anethum graveolens* leaves against the gram-negative bacteria *E. coli*.

## Materials and methods

The study was done at the Department of Biochemistry, Allahabad University, Allahabad, India. A botany expert from the institute authenticated the plants. Leaves of *Apium graveolens*, *Petroselinum crispum*, *Cichorium endivia,* and *Anethum graveolens* plants were used in the study. All experiments were performed 11 (n) times, based on data from previous studies and getting the maximum number. Leaves were crushed to form the extract. Further, bacterial culture plates were prepared, which were inoculated by *E. coli*. Furthermore, plant leaf extracts were added to the culture plates for diffusion studies. The inhibition of the growth of bacteria (presence or absence of a zone of inhibition) due to the plant extracts indirectly predicts its antibacterial efficacy [17].

Plant extract preparation

Ten percent leaf extracts of endive, celery, and parsley were prepared by separately pulverizing (crushing) 1 g each of the lyophilized leaves of the plant sample in a bone-china mortar pestle. The pulverized material was transferred to a 15 ml falcon tube, and 10 ml of different solvents (water, ethanol, isopropanol, and methanol) were next added and vortexed vigorously for 10 minutes and stood overnight. The samples in each case were next centrifuged (15 minutes, 6000 rpm) and then passed through a Whatman filter paper grade 1 to obtain the extract (10%, w/v). Plant extracts prepared in this way were stored in a refrigerator till the next day. In the case of dill, fresh leaves were washed, patted dry on filter paper, and air-dried. They were next processed for extract preparation as described above for other plants.

Preparation of Luria Bertani (LB) broth, Miller

6.25 g LB powder in 250 ml distilled water (2.5% w/v) was mixed and heated to dissolve it completely (if necessary). Finally, the solution was autoclaved at 15 lbs pressure (121°C) for 15 minutes.

Preparation of bacterial culture plates using Luria Agar (LA)

Twenty to 25 ml of autoclaved LA (1.6% w/v) was poured into each sterile plate and allowed to solidify in 30-60 minutes. All the antibacterial screening works were performed near the working zone of the flame (burner) to avoid any possible contaminations.

Preparation of bacterial culture

With the help of a sterilized wire loop, the bacterial colony was taken from the master plate and then transferred into a sterilized falcon tube containing 5 ml autoclaved LB solution. The falcon tubes that contain the bacterial suspension were vortexed to mix uniformly and incubated at 37°C for 24 hours. After 24 hours, we observed the falcon tubes with the naked eye, and the turbidity confirmed the bacterial growth. To again confirm the sterile zone of flame, blank 5 ml of LB solution was also incubated along with inoculated tubes. The grown bacterial culture was used for further experiments.

Antibacterial screening of herbs

The research employed diffusion methods due to their practicality and cost-effectiveness. Both well diffusion and disc diffusion methods were used. The disc diffusion method is considered to have better specificity, whereas the well diffusion method has better sensitivity. Moreover, the disc diffusion method shows activity at lower doses, whereas the well diffusion method shows antimicrobial activity at higher doses. Furthermore, the disc diffusion method is cheaper and much more convenient than the well diffusion method [18].

Well diffusion method

One hundred microliter bacterial inoculums were spread on Petri plates by using a spreader. Wells of 6 mm were made by sterilizing the tip (200 µl), and then 80 µl of different plant extract samples were added to respective wells. Finally, the plates were left at room temperature for 30 minutes for proper diffusion of extracts and then incubated at 37°C for 24 hours.

Disc diffusion method

One hundred microliter bacterial inoculums were spread on Petri plates by using a spreader. Autoclaved paper discs of 6 mm were dipped separately in different plant extract samples. After two minutes, with the help of sterilized forceps, dipped paper discs (plant extract containing) were transferred to the respective positions on the bacterial plate carefully. Finally, the plates were left at room temperature for 10 minutes and then incubated at 37°C for 24 hours.

## Results

As depicted in Table 1, various organic solvents yielded extracts with different colors, indicating the presence of different phytochemical compounds.

**Table 1:** Physical appearance (color) of all leaf extracts

Herbs	Water	Ethanol	Methanol	Isopropanol
*Apium graveolens* (celery)	Pale yellow	Light green	Forest green	Dark forest green
*Petroselinum crispum* (parsley)	Pale yellow	Lime green	Army green	Dark green
*Cichorium endivia* (endive)	Pale yellow	Fern green	Yellow	Moss green
*Anethum graveolens* (soya (dill))	Pale yellow	Olive green	Mint green	Forest green

Notably, our investigation showed a notable absence of antibacterial activity in the aqueous extract of all plant samples leading to the absence of a zone of inhibition (0 mm) (Table 2). Conversely, extracts using other solvents showed varying degrees of antibacterial activity, as evidenced in Table 2. Most of the zones of inhibition in the disc diffusion method were negative or slightly positive, which shows that the herbs studied are not very effective at lower doses (Table 2). The discovered false-negative findings underline the delicate interaction between solvent features and phytochemical properties, leading to the nuanced interpretation of antibacterial effects.

**Table 2:** Antibacterial activity of the leaf extracts by disc diffusion method For E and S, only the mean has been represented for simplicity of data, but for C, both the mean and standard deviation have been represented for comparison. The mean calculated value (C) of each plant in respective solvents was compared to the calculated zone of inhibition of penicillin (8±0.7 mm). C: calculated value; E: observed extract value; S: observed solvent value; x̄': mean; SD: standard deviation; *: p≤0.05=significant; **: p≤0.001=highly significant

Herbs	Calculated zone of inhibition (in mm)										
	Water		Ethanol			Methanol			Isopropanol		
	C	E-S	C (E-S) (x̄'±SD)	E (x̄')	S (x̄')	C (E-S) (x̄'±SD)	E (x̄')	S (x̄')	C (E-S) (x̄'±SD)	E (x̄')	S (x̄')
*Apium graveolens* (celery)	0		2±0.1**	12	14	-1±0.1**	8	9	-1±0.1**	10	11
*Petroselinum crispum *(parsley)	0		0±0.3**	14	14	0±0.2**	9	9	2±0.4**	13	11
*Cichorium endivia* (endive)	0		-6±0.1**	8	14	-3±0.1**	6	9	-2±0.6**	9	11
*Anethum graveolens* (soya (dill))	0		0±0.2**	14	14	-2±0.03**	7	9	-2±0.1**	9	11

Notably, the isopropanol extract of endive phytochemicals showed the highest zone of inhibition at 17 mm as compared to celery and soya phytochemicals (3 mm and 4 mm, respectively) in the well diffusion method (Table 3). The most favorable outcomes were observed with the isopropanol endive extract (17 mm) and the ethanolic soya extract (13 mm) (Table 3).

**Table 3:** Antibacterial activity of the leaf extracts by well diffusion method For E and S, only the mean has been represented for simplicity of data, but for C, both the mean and standard deviation have been represented for comparison. The mean calculated value (C) of each plant in respective solvents was compared to the calculated zone of inhibition of penicillin (14±1.1 mm). C: calculated value; E: observed extract value; S: observed solvent value; x̄': mean; SD: standard deviation; *: p≤0.05=significant; **: p≤0.001=highly significant

Herbs	Calculated zone of inhibition (in mm)										
	Water		Ethanol			Methanol			Isopropanol		
	C	E-S	C (E-S) (x̄'±SD)	E (x̄')	S (x̄')	C (E-S) (x̄'±SD)	E (x̄')	S (x̄')	C (E-S) (x̄'±SD)	E (x̄')	S (x̄')
*Apium graveolens* (celery)	0		-4±0.4**	8	12	0±0.3**	9	9	3±0.2**	13	10
*Petroselinum crispum* (parsley)	0		-2±0.6**	10	12	4±0.7**	13	9	-1±0.6**	9	10
*Cichorium endivia* (endive)	0		2±0.2**	14	12	1±0.6**	10	9	17±0.9**	27	10
*Anethum graveolens* (soya (dill))	0		13±0.3*	25	12	1±0.4**	10	9	4±0.4**	14	10

In the well diffusion method, as shown in Table 3, the aqueous leaf extract of all herbs has shown no antibacterial effect against *E. coli*, leading to the absence of a zone of inhibition (0 mm) (Table 3). Specifically, the ethanolic extract of endive and soya phytochemicals showed a positive zone of inhibition measuring 2 mm and 13 mm, respectively. However, for celery and parsley, the interaction with ethanol appeared to interfere with its physiochemical properties, resulting in calculated false-negative zones of inhibition (-4 mm and -2 mm, respectively) (Table 3) [19].

In contrast, the methanolic extract of parsley phytochemicals showed the maximum zone of inhibition at 4 mm as compared to other solvents, while endive and soya phytochemicals showed a relatively smaller zone of inhibition at 1 mm (Table 3).

The antibacterial effectiveness of blank solvents followed a decreasing order, ethanol>isopropanol>methanol>water, in both well and disc diffusion methods (Table 4).

**Table 4:** Antibacterial activity results of the blank solvents x̄': mean; SD: standard deviation; *: p≤0.05=significant; **: p≤0.001=highly significant

Solvents	Observed zone of inhibition (in mm)					
	Well diffusion			Disc diffusion		
	Solvent (x̄'±SD)	Penicillin (x̄'±SD)	P-value	Solvent (x̄'±SD)	Penicillin (x̄'±SD)	P-value
Water	0±0.1	14±1.1	0.001**	0±0.2	8±0.7	0.001**
Ethanol	12±0.4	14±1.1	0.001**	14±1.1	8±0.7	0.001**
Methanol	9±0.6	14±1.1	0.001**	9±0.5	8±0.7	0.02*
Isopropanol	10±0.7	14±1.1	0.001**	11±2.1	8±0.7	0.001**

The antibacterial efficacy of penicillin (antibiotic) in both well and disc diffusion methods was done at a concentration of 1 mg/ml, and the zones of inhibition were found to be 14 mm and 8 mm, respectively. The minimum inhibitory concentration (MIC) value of penicillin was 0.1-1 µg/ml [20].

## Discussion

Phytochemicals with chemoprophylactic properties include alkaloids, flavonoids, tannins, terpenoids, glycosides, saponins, and anthraquinones. The nitrogenous compounds in alkaloids give antimicrobial properties to the plants. Antioxidant or free radical scavenger properties shown by flavonoids and phenolics are the most important properties of plant phytochemicals. Phytochemicals show many functions, notably the main role in plant defense against pathogens and predators, which also control human pathogenic infections [21].

The rise and development of antibiotic resistance provide a significant and alarming danger to human health, urging an immediate need for newer solutions. Unfortunately, the rate of development of novel medicines is constrained by various challenges, and progress in this area is often slow [2]. Given these concerns, our study aimed to contribute to the ongoing efforts in combating antimicrobial resistance by exploring the antibacterial potential of specific culinary herbs.

The research employed diffusion methods due to their practicality and cost-effectiveness. Notably, our investigation shows a notable absence of antibacterial activity in the aqueous extract of all plant samples. This finding occurs due to improper extraction. Another possible reason is the presence of precipitates in all aqueous extracts. These precipitates, owing to their instability, may hinder the proper diffusion of phytochemicals in the well or disc agar plates, compromising the antibacterial activity.

The observed extent of the zone of inhibition depends upon the antibacterial potency and diffusing ability of the components within the extract [19]. Variability in phytochemical composition may account for the distinct inhibitory effects exhibited by the same herbs in different solvent extracts. These findings highlight the nuanced relationship between solvent choice, phytochemical extraction, and resultant antibacterial activity.

The absence of a zone of inhibition in the disc method may be due to improper extraction of phytochemicals or due to the solvent's effect. In the published literature, it has been reported that false-negative antibacterial results in disc diffusion methods are due to the polar nature of phytochemicals. These characteristics may further be due to intermolecular forces, particularly the hydrogen bonding capability of cellulose (paper), influencing the retention or absorption of phytochemicals on the disc surface and subsequently impeding their diffusion [22]. The possibility that phytochemicals are polar compounds may explain the observed interference with diffusion in the disc diffusion method. The disc diffusion method may exhibit lower sensitivity to natural products when compared to the well diffusion method.

The most favorable outcomes were observed in the well diffusion method, with the isopropanol endive extract (17 mm) and the ethanolic soya extract (13 mm). From these results, we can infer that isopropanol is a suitable solvent choice for extracting endive phytochemicals, while ethanol is preferable for soya. Further, the antibacterial activity results of the blank solvents were also estimated, to assess any contributory effect of solvants on the antibiotic activity of leaf extracts. Phytochemicals in endive and soya exhibit antibacterial effects that closely resemble the performance of the antibiotic penicillin (1 mg/ml). While penicillin is administered in pure form, the extract from endive and soya, being impure, shows an antibacterial effect comparable to the antibiotic.

This promising finding underscores the potential of endive and soya as alternative sources for natural antibiotics in the future. Further investigation will be imperative to investigate and characterize the specific antibacterial compounds present in endive and soya, a good alternative to natural sources of antibiotics against human diseases. Our study contributes to this endeavor by shedding light on the antibacterial potential of certain culinary herbs, providing a foundation for further research into harnessing their benefits for human health. These promising results encourage continued exploration of natural sources for innovative antibacterial formulations, striving towards a sustainable and effective approach to combating infectious diseases.

Study limitations

Mechanical methods were used in leaf extract preparation. This may add subjective variability in preparations. Also, only one antibiotic was used in the study.

## Conclusions

The systematic screening of leaf extracts from select culinary herbs to evaluate their antibacterial efficacy against *E. coli* has yielded promising and noteworthy outcomes. Among the herbs investigated, organic extracts of endives and soya have emerged as particularly potent sources of antibacterial activity as compared to the other herbs. This study lays the foundation for the future focused on isolating novel phytochemical compounds from endives and soya, with the aim of replacing current antibiotics that may carry toxic or harmful chemicals. Our findings underline the limitations of aqueous extracts in exhibiting antibacterial effects, indicating the importance of using more effective solvents for extracting phytochemicals from these herbs. Future attempts should involve isolating and screening these phytochemical compounds against various bacterial strains, including gram-positive bacteria, to completely assess their antibacterial potential.

Notably, the selected herbs not only show promise as potential antibiotics but also offer eco-friendly, biocompatible, and non-toxic attributes. The implications of this study extend beyond the realm of antibacterial efficacy, positioning these herbs as versatile agents with a multitude of applications across different domains.
